# Geometric morphometric analysis of Japanese female facial shape in relation to psychological impression space

**DOI:** 10.1016/j.heliyon.2020.e05148

**Published:** 2020-10-02

**Authors:** Koyo Nakamura, Anri Ohta, Shoko Uesaki, Mariko Maeda, Hideaki Kawabata

**Affiliations:** a Faculty of Science and Engineering, Waseda University, Japan; b Japan Society for the Promotion of Science, Japan; c Keio Advanced Research Centers, Japan; d R&D, Sunstar Inc., Takatsuki, Osaka, Japan; e Department of Psychology, Faculty of Letters, Keio University, Japan

**Keywords:** Psychology, Facial impression, Facial shape, Geometric morphometric analysis, Female face

## Abstract

Facial appearance has essential consequences in various social interactions. Previous studies have shown that although people can perceive a variety of impressions from a face, these impressions may form from a relatively small number of core dimensions in the psychological impression space (e.g., valence and dominance). However, few studies have thus far examined which facial shape features contribute to perceptions of the core trait impression dimensions for Asian female faces. This study aimed to identify the commonalities between various facial impressions of Japanese female faces and determine the facial shape components associated with such impressions by applying geometric morphometric (GMM) analysis. In Experiment 1 (Modeling study), Japanese female faces were evaluated in terms of 18 trait adjectives that are frequently used to describe facial appearance in daily life. We found that Japanese female facial appearance is indeed evaluated mainly on the valence and dominance dimensions. In Experiment 2 (Validation study), we confirmed that all the trait impressions were quantitatively manipulated by transforming the facial shape features associated with valence and dominance. Our results provide evidence that various facial impressions derived from these two underlying dimensions can be quantitatively manipulated by transforming facial shape using the GMM techniques.

## 1 Introduction

Facial appearance is one of the salient sources of personal information that strongly affects various social interactions. People judge sociodemographic characteristics such as gender, ethnicity, and age from facial appearance with high accuracy [1, 2] and form various trait impressions based on it without conscious effort [3]. People often believe that some internal traits or personality aspects can be inferred from facial appearance [4], though this is not necessarily accurate [5, 6]. Nevertheless, facial impressions have essential social consequences in wide-ranging social interactions, including mate choice [7], hiring decisions [8], elections [9, 10, 11], and even science communication [12]. Given these real-life consequences, psychologists have tried to investigate various aspects of facial appearance such as attractiveness, trustworthiness, dominance, and competence and to identify which facial features make a face attractive, dominant, or socially desirable [13, 14, 15]. Perceptions of these facial impressions are widely shared across different populations and cultures [16, 17, 18, 19], and the ability to form such impressions can be acquired early in development [20, 21, 22], indicating the universal and inherent nature of such perceptions.

Although people can perceive a variety of impressions from a face, these impressions are often highly correlational [13, 14, 16, 23]. For example, attractiveness positively correlates with trustworthiness, whereas dominance positively correlates with threat and masculinity [14]. This raises the possibility that there are commonalities behind a variety of facial impression evaluations. In other words, facial impressions may form from a relatively small number of dimensions in the psychological impression space [13]. In the past decade, independent studies have identified some fundamental dimensions underlying several types of facial impressions [13, 14, 16, 24]. An early study performed principal component analysis (PCA) to reduce a wide variety of facial trait ratings (e.g., trustworthiness, dominance, competence, attractiveness) to a small number of orthogonal dimensions, demonstrating that only two impression dimensions, *valence* (emotionally negative-positive) and *dominance* (obedient-dominant), are enough to account for over 70% of different facial impressions [14]. The authors argued that valence is thought to reflect whether the person's intentions are emotionally positive or negative toward the perceivers (e.g., approachability, trustworthiness, and attractiveness), whereas dominance is thought to reflect the person's ability to act upon those intentions (e.g., dominance, threat, and masculinity) [14]. From an evolutionary psychology perspective, valence could relate to recognizing reliable cues of mate value and reproductive success [25], while dominance could predict physical strength [26, 27]. Similar results were independently obtained by later studies that identified three core dimensions, *approachability*, *dominance*, and *youthful-attractiveness*, and indicated that various facial impressions may be derived from these dimensions [16, 28].

These core dimensions of the psychological impression space have so far mainly been examined in Western perceivers' impressions of Caucasian faces, except for one recent cross-cultural study that found substantial cross-cultural agreement in some dimensions of Western and Asian perceivers' impressions of Caucasian and Asian faces [16]. Impression dimensions such as approachability and youth-attractiveness were found to be shared between Western and Asian raters, suggesting that, even if perceptions of facial impressions are shaped by culture-specific cues, the dimensions underlying various impressions can function in similar ways across cultures [16]. This was the first study to indicate the universality of impression dimensions, where *ambient images*, photographs that are highly different in aspects such as lighting, angle, and facial expressions, were used. While the use of ambient images is beneficial in capturing naturally occurring facial variations in everyday life, well-controlled facial stimuli would enable the identification of subtle features driving the principal dimensions of facial impressions.

One alternative facial metric is provided by geometric morphometric (GMM) analysis. GMM is a powerful tool for demonstrating variations of physical facial shape characteristics that are locally and globally associated with specific biological and psychological factors without imposing any *a priori* constraints [29]. GMM is based on a multivariate analysis of the Cartesian coordinates of facial landmark points and seeks to identify and quantify the associations between the relative spatial positions and the variables of interest. Using GMM analysis, previous studies have quantified morphological cues to various impressions, such as attractiveness [30, 31, 32, 33], trustworthiness [34, 35], dominance [33], aggressiveness [36], intelligence [37], and sexual dimorphism [38, 39]. These studies have demonstrated that different impressions are perceived from the unique combination of multivariate facial features. For attractiveness, which is highly correlational to valence, faces with bigger eyes, larger eyebrows, a mouth with upward-pointing corners, and a generally extended and narrower shape are perceived as being more attractive [33]. For aggressiveness, which is highly correlational to dominance, faces with downturned eyebrows are perceived as more aggressive [36].

Faces vary along multiple dimensions, and these shape variations and their combinations can induce different facial impressions. However, few studies have thus far examined which facial shape features contribute to perceptions of the core trait impression dimensions for non-Western faces. Thus, we aimed to identify the commonalities in various impressions of Japanese female faces and the facial shape components associated with such dimensions by using GMM analysis. In the present study, we collected photographs of female faces and asked a different set of participants, the female raters, to evaluate their facial impressions of the photographs. We investigated which fundamental dimensions of facial impressions could be obtained from Japanese female faces and compared the results to those of previous studies [13, 14, 16] using PCA by evaluating facial impressions. In this analysis, we investigated the relationships between the facial shapes of the photographs and the resulting facial impressions. Second, we performed landmark-based GMM analysis, which can statistically analyze and visualize the shape information of anatomical structures to correlate the main dimensions of facial impressions (Modeling study). Finally, we validated the GMM results using a rating experiment in which independent raters indicated their impressions in response to composite facial images made as a function of the dimensions revealed by the GMM (Validation test).

## 2 Experiment 1: modeling study

### 2.1 Methods

#### 2.1.1 Participants

Twenty-four Japanese female participants were recruited as raters through a research company (mean age = 49.00, *SD* = 5.98, range 40–58). All participants had normal or corrected-to-normal vision and were naive to the purpose of the study. All participants provided written informed consent. The protocols of the study were in accordance with the Declaration of Helsinki and were approved by the ethics committee of Keio University.

#### 2.1.2 Acquisition of facial photographs

We collected frontal photographs from 102 models who were ethnically Japanese females (mean age = 41.74, *SD* = 8.84, range 30–64), none of whom were raters, recruited from the research company, and gave their written informed consent. Acquisition of facial photographs was approved by the ethics committee of Keio University. All models were naive to the purpose of this study. All photographs were taken using a digital reflex camera (Canon EOS 7D) with a 128 mm lens, electronic flash, and reflection screen on a white background. To obtain natural and non-smiling expressions, the models were seated and viewed a screen on which neutral images from the International Affective Picture System [40] were presented at a distance of 4 m. The camera was positioned in a hole in the screen at approximately the models' eye height. We chose to avoid facial cosmetics, accessories, and other facial decorations as much as possible. For the rating experiment, the photographs were masked to obscure hair and clothes, and their image size was reduced to a rectangle of 307 × 384 pixels.

#### 2.1.3 Image ratings

For composing scales to rate facial impressions in the experiment, we conducted preliminary investigation as follows. Initially, five individuals (two women and three men) made a list of Japanese adjectives related to attitudes, feelings, personality, and values concerning female facial impressions. A total of 78 adjective items were listed, including sutekina (corresponding to ‘lovely’ in English), seijitsuna (conscientious), jiritsushita (autonomous), irokenoaru (amorous), and so on, and then we asked 62 women to select 15 items based on which they care about how others see themselves and to select 15 items based on which they want others to see, using a paper survey. Each participant was allowed to select the same items between these two types of selection. Based on the results of this survey, we selected the following Japanese items to rate female facial impressions in the order of the amounts of selections, taking into account the two main indicators of valence and dominance: ikiiki (corresponding to ‘lively’ or ‘agile’ in English), iraira (irritated), kawaii (cute), kirei-na (beautiful), keizaiteki-ni-yoyu-ga-ari-sou (financially fit/rich), kokoro-ga-hiro-sou (open-minded), sabasaba-shi-te-sou (not niggled), shakoteki-na (sociable), johin-na (graceful), seiketsukan-ga-ari-sou (fresh/clean), tano-shi-sou (cheerful), tayo-ri-ni-nari-sou (reliable/trustworthy), chiteki-na (intelligent/knowledgeable), tsukare-te-i-sou (tired), ningenmi-ga-ari-sou (humanly), hana-shikake-yasu-sou (approachable), mawa-ri-ni-naga-sa-reyasu-sou (easily influenced), and rin-to-site-iru (dignified).

In this experiment, the 24 raters individually viewed the set of 102 female faces presented on a laptop computer (ASUS VivoBook X200CA) at a 1366 × 768 spatial resolution from a distance of approximately 40 cm. They rated their impressions of the faces on a visual analog scale ranging from 0 (least) to 100 (most) positioned beneath each image. The images were presented until participants made their responses. The order of the faces was fully randomized, and the order of impressions to be rated was counterbalanced across participants.

#### 2.1.4 Geometric morphometric analysis

Using GMM, we tried to identify the facial configurations associated with the perceived dimensions underlying a variety of impressions. The GMM analysis comprised a multivariate statistical analysis of facial shape. First, to represent the facial shapes of the Japanese female faces, a total of 72 landmarks (including 36 semilandmarks) [41] placed on each face were digitized using tpsDig2 software (version 2.26) [42] and vectorized in two-dimensional space. The landmark locations on the faces were adopted from previous studies [34, 41], in which landmarks are represented as points that are geometrically homologous in different individuals while semilandmarks denote curves and outlines. For precise localization of the landmarks, two independent technical collaborators with no knowledge of the purpose of the study placed the landmarks manually. To make sure the landmarks the collaborators placed were consistent, we calculated a 2D correlation coefficient for each face, and extremely high positive correlation coefficients were obtained (average *r = 0.99*, range = [0.997–0.999]). Therefore, the average positions of the two collaborators' landmarks were used in the analyses. In addition, 36 semi-landmarks were slid by tpsRelw software (version 1.65), and the 66 landmark configurations were symmetrized by averaging each configuration with its relabeled reflection [33, 39, 43]. All the landmark and semi-landmark configurations were subsequently superimposed by generalized Procrustes analysis in the tpsRelw to standardize the face size and optimize rotation and translation, thereby minimizing the distances between corresponding landmarks. Next, a principal component analysis (PCA) was carried out to translate the original shape coordinates (i.e., 72 superimposed landmark coordinates per face) into orthogonal principal components (i.e., a total of 101 PCs). We then selected the first 14 PCs, which accounted for more than 95% of the face variation. To identify any statistically significant associations between face impression dimensions and facial shapes, we performed a permutational multivariate regression analysis of variance using distance matrices with 9,999 permutations implemented in the *adonis* function of *Vegan* in *R* (Version 3.5.1)^1^. We ran a multiple multivariate regression with the 14 facial shape PC scores as the response variable and with the facial impression scores extracted via PCA for facial impression ratings as the explanatory variables; these are explained in detail later.

### 2.2 Results & discussion

#### 2.2.1 Commonalities among the 18 facial impressions

**Figure 1:**
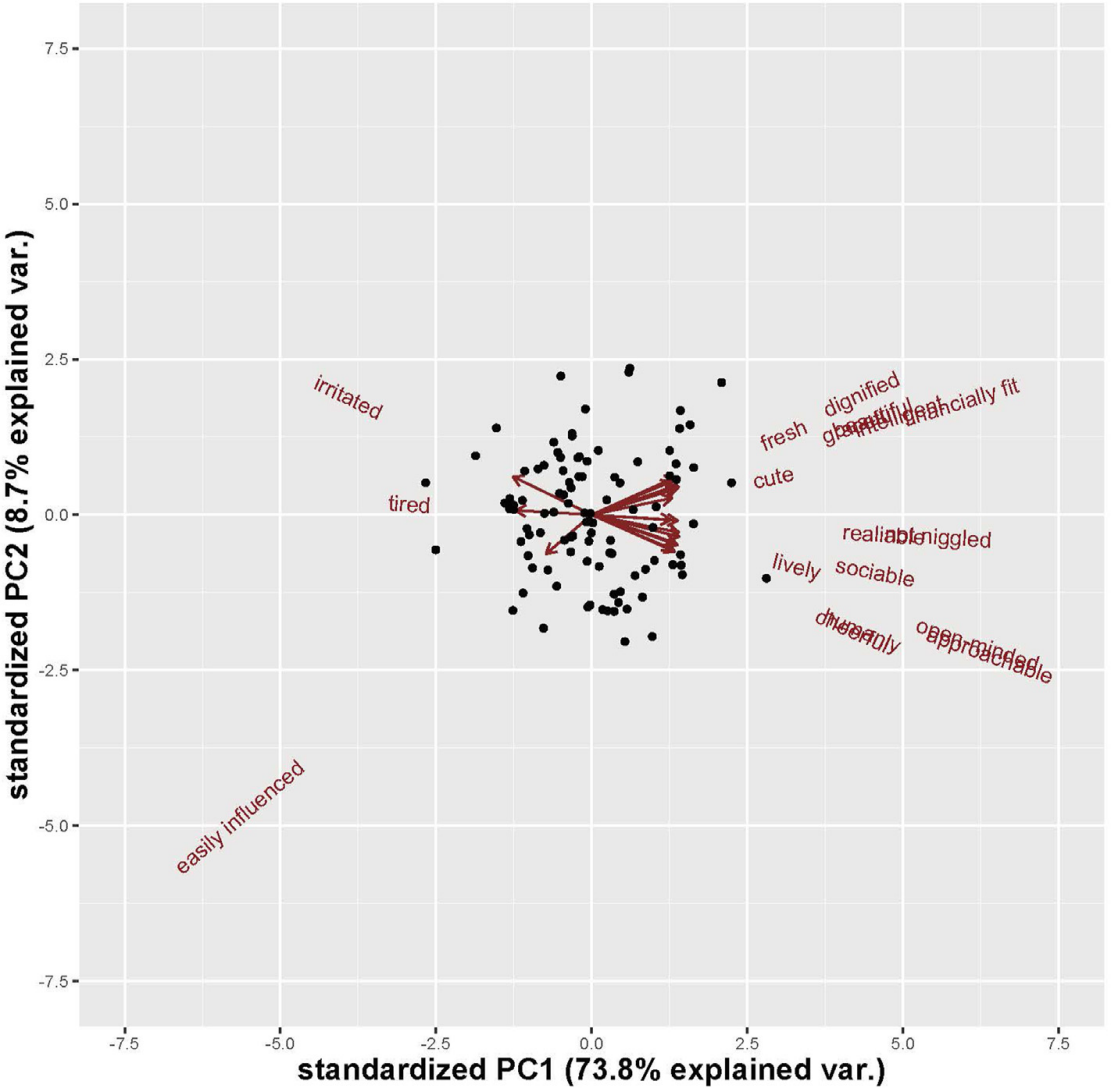
Biplot of the principal components extracted from PCA of 18 female facial impressions. Each dot represents a single female face.

We analyzed the various impression ratings to identify the commonalities among the 18 facial impressions. In the first step, we calculated Cronbach's alphas for all impression ratings, confirming that all 18 ratings were highly reliable across participants (all Cronbach's alphas >.77). The mean ratings across participants were then computed and used thereafter. In the second step, we entered the mean 18 facial impression ratings for the 102 faces into PCA. The first principal component (PC1) accounted for 73.8% of the variance, and the second (PC2) accounted for 8.7% (Figure 1). Although the rest of each PC accounted for less than 6.5% of the variance, we took the first two PC into account hereafter. The PC loadings of all the impressions are shown in Table 1. From the observation that emotionally positive valence ratings (e.g., *lively*, *sociable*, *intelligent*, etc.) had positive loadings and emotionally negative valence ratings (e.g., *irritated*, *tired*, and *easily influenced*) had negative loadings on PC1, this component can be interpreted as an emotional valence dimension. Likewise, as the ratings of *fresh*, *graceful*, and *dignified* had positive loadings and *cheerful*, *humanly*, and *open-minded* had negative ones on PC2, it can be interpreted as a dominance dimension. In the following section, we aimed to identify the facial shape components associated with the valence and dominance dimensions obtained from the above-mentioned PCA.

**Table 1:** PCA loadings of facial impression ratings.

Items to be rated	PC1	PC2
lively/agile	0.25	-0.19
sociable	0.25	-0.15
intelligent/knowledgeable	0.25	0.24
reliable/trustworthy	0.25	-0.05
approachable	0.25	-0.25
beautiful	0.25	0.25
financially fit/rich	0.24	0.22
dignified	0.24	0.32
open-minded	0.24	-0.24
graceful	0.24	0.24
cheerful	0.24	-0.31
humanly	0.23	-0.29
cute	0.23	0.14
fresh/clean	0.23	0.28
not niggled	0.23	-0.04
easily influenced	-0.13	-0.33
tired	-0.22	0.04
irritated	-0.23	0.32

Note. PCA loadings were calculated by the PCA applied to the facial impression ratings that were aggregated across raters.

#### 2.2.2 Facial shape components associated with two underlying facial impression dimensions

**Figure 2:**
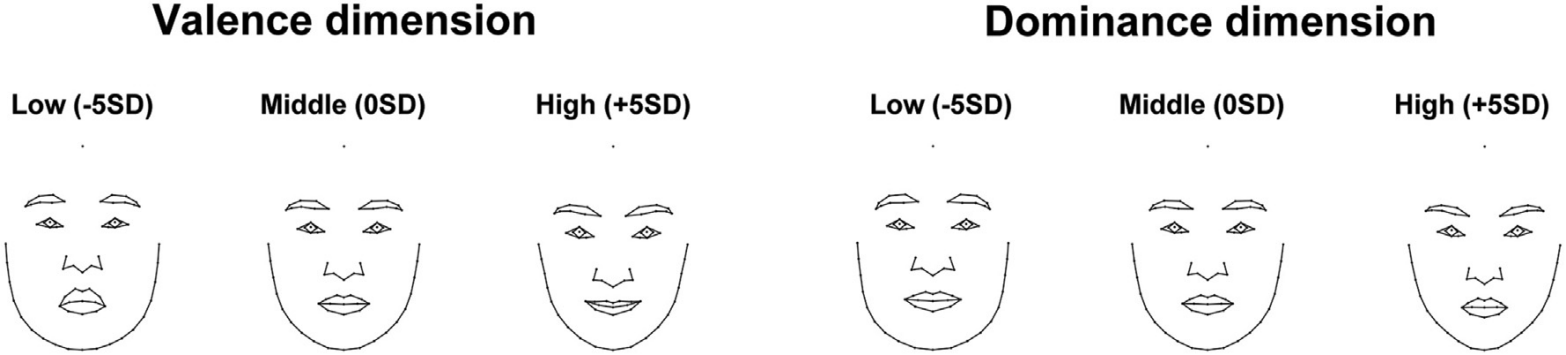
Female facial shape changes with the valence and dominance dimensions. Compared to the average female face (Middle), a facial shape with low valence/dominance (Low) and one with high valence/dominance (High) are visualized. The extent of the changes is presented in SD units.

To identify the associations between facial shape components and the two underlying facial impression dimensions, we performed multivariate regression analysis for the first 14 PCs obtained from facial landmark variations with the valence (PC1) and dominance (PC2) scores as independent variables. This revealed significant relationships between multivariate facial shape components and the valence dimension on the one hand (*F*(1,101) = 8.75, *p* < .001, R^2^ = .08) and the dominance dimension on the other (*F*(1,101) = 2.73, *p* = .01, R^2^ = .02). These two effects were still statistically significant even after controlling for the age of the models (*ps* < .05). Figure 2 shows the visualization of shape regression on the valence and the dominance dimension.

## 3 Experiment 2: validation test

To validate the GMM results, we created shape-transformed average faces along the valence and dominance dimensions. We tested whether transforming the facial shape components associated with the valence and dominance dimensions would change the impressions evaluated using the 18 adjectives.

### 3.1 Methods

#### 3.1.1 Participants

Twenty-six Japanese female participants were recruited from Keio University. None of the participants took part in the Modeling study (mean age = 21.76, *SD* = 1.50). All participants had normal or corrected-to-normal vision and were naive to the purpose of the study. The protocols of the study were in accordance with the Declaration of Helsinki and were approved by the ethics committee of Keio University.

#### 3.1.2 Stimuli

**Figure 3:**
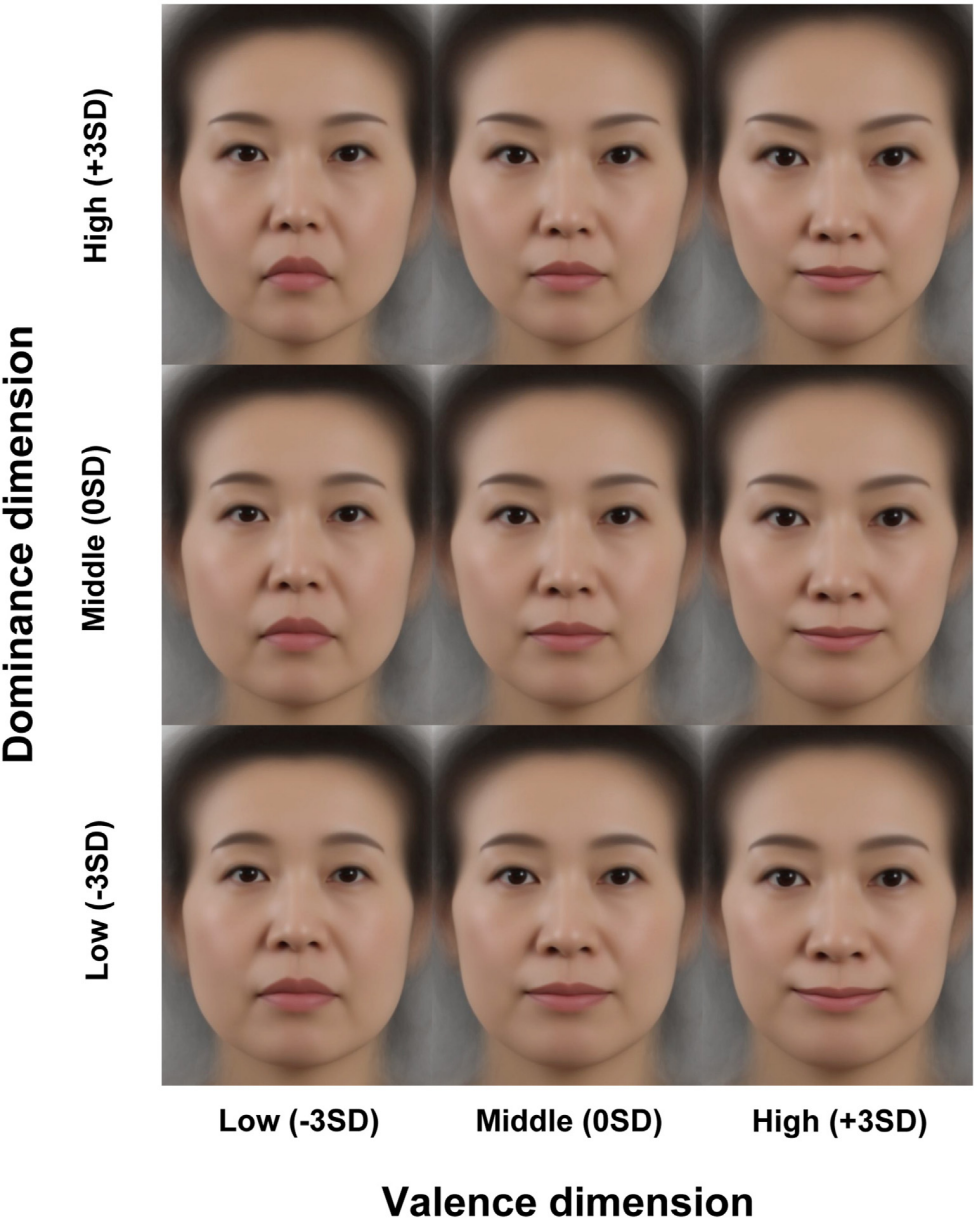
Female face images used in Experiment 2 (Validation study). The average female image made from 102 models collected in the Experiment 1 was independently unwarped along the valence and dominance dimensions. The nine versions of faces at three valence and dominance levels were generated and rated in terms of 18 Japanese adjectives.

We created nine versions of morphed average faces from the 102 models that differed in the valence (negative [-3SD] vs. neutral [0SD] vs. positive [+3SD]) and dominance dimensions (non-dominant [-3SD] vs. neutral [0SD] vs. dominant [+3SD]) by unwarping each of the original average face images along each of the transformed target landmark configurations (Figure 3) using tpsSuper (version 2.05). The faces were presented as 600 × 800 images on an LCD display with a 1920 × 1080 resolution and a vertical refresh rate of 60 Hz, and they were viewed at 60 cm from the display. The experiment was performed using Psychopy (version 3.1) [44], a python library for conducting psychological experiments.

#### 3.1.3 Procedure

The participants were required to rate 18 impressions for the nine versions of transformed average faces, including an original average face. The 18 impression adjectives were identical to those used in the Modeling study, and each was evaluated on a visual analog scale ranging from 0 to 100. The rating task was divided into nine blocks, with one of the nine faces presented for four seconds and the 18 impressions rated in a row. The order of the nine blocks and 18 impressions was randomized across participants.

#### 3.1.4 Linear mixed effects model

The rating scores for each impression were analyzed with a linear mixed effect model (LME) using the *lmer* function in the *lme4* package for *R* [45]. The valence and dominance dimension values (in SD) were entered as within-subject factors and treated as continuous variables. In the model, an intercept and random slopes for valence and dominance dimension values were included in the participants predictor.

### 3.2 Results & discussion

Table 2 summarizes the regression coefficients of the LME model on the 18 impression ratings. The *p* value <0.05 was considered statistically significant. Bonferoni correction for multiple comparisons was applied dividing the *p* value by the number of comparisons made, separately for the valence and dominance dimensions. All the regression coefficients of valence were statistically significant (*ps* < .003), except for *humanly* (*p* = .11), whereas the regression coefficients of dominance were statistically significant for all impressions except *lively*, *cute*, *financially fit*, *sociable*, *graceful*, *fresh*, *reliable*, *intelligent*, *tired*, and *dignified* (*ps* > .05).

**Table 2:** Regression coefficients of valence and dominance in LME.

	Intercept	Valence (PC1)	Dominance (PC2)
lively/agile	46.86	6.55 ∗∗∗	-0.13
irritated	40.63	-3.51 ∗∗∗	3.31 ∗∗∗
cute	40.12	5.62 ∗∗∗	1.06 ∗
beautiful	54.86	7.34 ∗∗∗	1.87 ∗∗∗
financially fit/rich	52.82	4.31 ∗∗∗	0.51
open-minded	47.07	3.84 ∗∗∗	-4.42 ∗∗∗
not niggled	54.91	2.37 ∗∗	2.23 ∗∗∗
sociable	51.98	5.80 ∗∗∗	-0.89
graceful	56.55	6.35 ∗∗∗	-0.02
fresh/clean	58.68	5.30 ∗∗∗	0.43
cheerful	40.76	6.53 ∗∗∗	-1.73 ∗∗
reliable/trustworthy	54.40	6.17 ∗∗∗	-0.52
intelligent/knowledgeable	53.89	5.46 ∗∗∗	1.49 ∗
tired	51.60	-6.13 ∗∗∗	-0.68
humanly	55.99	1.00	-2.58 ∗∗∗
approachable	48.45	3.70 ∗∗∗	-3.78 ∗∗∗
easily influenced	39.45	-3.49 ∗∗∗	-3.10 ∗∗∗
dignified	53.70	7.45 ∗∗∗	1.61 ∗

*Note.* ∗*p* < .05; ∗∗*p* < .01; ∗∗∗*p* < .001.

**Figure 4:**
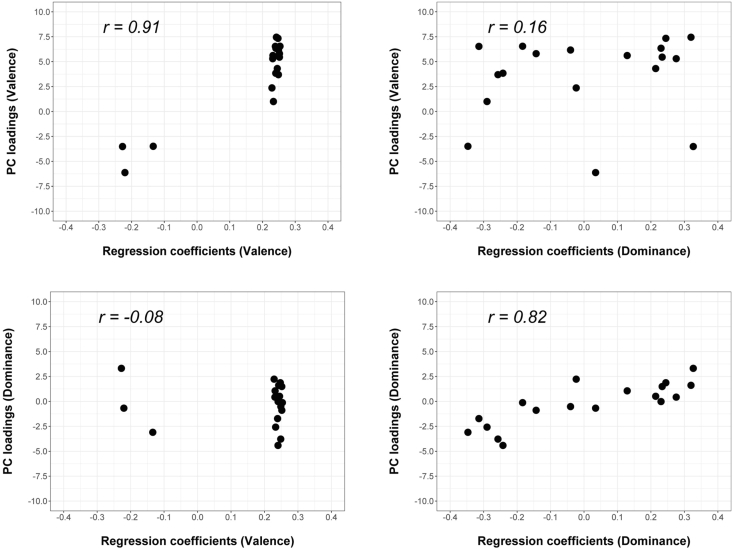
Pearson's correlation coefficients between the PC loadings of 18 impressions on the valence and dominance dimensions and the LME regression coefficients. Each dot indicates an impression evaluated in terms of 18 Japanese adjectives.

To further examine what determines the extent to which valence and dominance affect perceived impressions, we computed Pearson's correlation coefficients between the PC loadings of the 18 impressions on the valence and dominance dimensions and the LME regression coefficients (Figure 4) with Bonferoni correction. The results showed that the PC loadings on the valence dimension were positively correlated with the regression coefficients of valence manipulation (*r* = .91, *t*(16) = 8.90, *p* < .001, 95% CI [0.78–0.97]), but they were not correlated with the regression coefficients of dominance manipulation (*r* = -.08, *t*(16) = -0.32, *p* = .75, 95% CI [-0.53–0.40]). In contrast, the PC loadings on the dominance dimension were positively correlated with the regression coefficients of dominance manipulation (*r* = .82, *t*(16) = 5.82, *p* < .001, 95% CI [0.58–0.93]), but they were not correlated with the regression coefficients of valence manipulation (*r* = .16, *t*(16) = 0.68, *p* = .50, 95% CI [-0.33–0.58]). These results indicate that the extent to which impressions change by transforming the face along the valence and dominance dimensions largely depends on how much each impression relates to the two dimensions.

## 4 General discussion

This study aimed to identify the commonalities between various impressions of Japanese female faces and determine the facial shape components associated with the dimensions of these impressions by using GMM analysis. In this study, Japanese female faces were evaluated in terms of 18 impression adjectives that are frequently used to describe facial appearance in daily life. We found that Japanese female facial appearance is evaluated mainly in terms of two fundamental impression dimensions: valence and dominance. Several previous studies on Caucasian facial impressions have shown that these two fundamental dimensions are robustly extracted from well-documented impressions such as attractiveness, trustworthiness, dominance, and competence [13, 14]. Furthermore, one recent cross-cultural study showed the universality of psychological impression space between Western and Asian perceivers [16]. The results from our study using common Japanese adjectives [46], were consistent with the previous findings that valence and dominance constitute the core impression dimensions. The use of common adjectives is advantageous to having raters clearly understand what it is they are evaluating. Indeed, the inter-rater reliability of all the impressions in this study was substantially high, indicating that the meaning of the adjectives was sufficiently understandable to all the raters.

Valence accounted for 73.8% of the variance in all impressions in this study, while dominance explained 8.7% of the variance. As such, almost 80% of all the impressions examined in this study were represented by these two fundamental dimensions. These results were consistent with previous research indicating that valence accounted for more than half of the variance in a set of Caucasian computer-generated male facial impressions [14]. Although the nature and number of the core impression dimensions is still being debated, our results showed that at least valence is a strong determinant of Japanese female facial impressions as well.

More importantly, we identified the facial shape components associated with valence and dominance by using GMM, which enabled us to relate the two with great precision and thereby reveal that both global configuration and local parts on faces affect valence and dominance. In particular, the valence dimension is characterized by structural features resembling a smile, such as an upward curving mouth and upturned eyebrows (Figure 1). These facial features signal whether to approach or avoid a person [6, 14, 47]. Judging approachability, which is analogous to valence, relies heavily on the shape of the mouth. As can be seen from Figure 1, the corners of the mouth lift up as the valence-related facial shape is exaggerated.

On the other hand, dominance is characterized by structural features resembling an angry expression, such as lower inner eyebrows and downturned corners of the mouth. Faces with such patterns are likely to be evaluated as angry and dominant [14, 47]. Valence and dominance may stem from adaptive responses to emotional expressions [48], as people are likely to approach a smiling person and avoid a person with an angry expression. Our GMM results support this view [6, 48]. Furthermore, the associations between facial components and the two impression dimensions were significant even after controlling for the age effect. This suggests that facial shape cues to valence and dominance can be isolated from aging cues [13].

Of particular note in this study is that we were able to quantitatively manipulate each of the 18 impressions based on the GMM results. Indeed, the validation test confirmed that facial shape transformation along the valence dimension affected 17 of the 18 impressions, and the degree of the effect was positively correlated with the PC loading of each impression onto the valence dimension. On the other hand, facial shape transformation along the dominance dimension affected 8 of the 18 impressions, and the degree of the effect was positively correlated with the PC loading of each impression on the dominance dimension. These results provide evidence that the transformation of facial shape components along the valence and dominance dimensions allows for the systematic manipulation of various impressions. That is to say, an arbitrary facial impression can be manipulated predictably by simply calculating how much the impression correlates with the valence and dominance dimensions as indexed by the PC loadings of the impression. As indicated in previous research, different impressions are highly correlational, so identifying the commonalities between impressions can offer practical benefits in manipulating facial impressions [14].

Despite some important findings in our study, there are several limitations to consider. First, while we used many trait adjectives, some commonly used in the study of Japanese facial impressions, to cover diverse impressions, a large proportion of the variance in the impressions was nevertheless explained solely by the valence dimension. As such, we cannot conclude whether this suggests Japanese female facial impressions are largely determined by the valence dimension or if the trait adjectives in this study do not cover the entirety of the variations of impressions for Japanese female faces. This might be partially because our study targeted only female facial impressions by female raters, while previous studies investigated both male and female faces [13, 14, 16]. Dominance-related impressions (e.g., dominance, masculinity, threat, and aggressiveness) are well-documented as critical determinants of male facial impressions [26, 27]. Although physical appearance (e.g., facial attractiveness) can be more important in women compared to men [49, 50], male impressions should be similarly examined and compared to the female ones [14, 33, 51]. Future studies should include both male and female faces and cover impressions such as social dominance and threat to gain a better understanding of the psychological impression space. Another limitation lies in the fact that the age of the photographic models and raters in Experiments 1 and 2 was unmatched. Although it is known that perceptions of facial impressions are consistent even among people of different ages [52], future studies should address whether the rater's age has modulating effects on perceptions of facial impressions. Despite these limitations, our findings provide important insights into the social perceptions of Japanese female faces and establish GMM-based parametric transformation of facial impressions.

A future approach of a GMM analysis on facial impressions will be further developed by recent advances in facial image processing [53], such as automatic facial feature detection [54] or computational models leveraging a deep neural network [55, 56]. Although we targeted trait impressions perceived from a static facial image in this study, yet newly developed computational models allow for quantifying a dynamic aspect of facial features (e.g., facial movements by expressing emotions) [57, 58]. Given that there is the interplay between the perception of facial impressions and expressions [47], a more refined model that integrates both static and dynamic aspects of facial shape driving the perception of trait impressions contributes to a deeper understanding of how people form impressions from facial appearance in the real world.

In conclusion, our study shows that first impressions of Japanese female faces are evaluated on the valence and dominance dimensions, and that various facial impressions derived from these two underlying dimensions can be quantitatively manipulated by transforming facial shape using the GMM techniques. This approach may lead to a deeper understanding of what facial features drive facial impressions through generating well-controlled facial images that vary in perceived impressions.

## Declarations

### Author contribution statement

K. Nakamura: Conceived and designed the experiments; Analyzed and interpreted the data; Wrote the paper.

A. Ohta, S. Uesaki, M. Maeda: Conceived and designed the experiments; Performed the experiments; Contributed reagents, materials, analysis tools or data.

H. Kawabata: Conceived and designed the experiments; Performed the experiments; Analyzed and interpreted the data; Wrote the paper.

### Funding statement

This work was supported by 10.13039/501100001697Keio University and Sunstar Co. Ltd.

### Competing interest statement

The authors declare the following conflict of interests: S. Uesaki, A. Ohta and M. Maeda who work at Sunstar Co. Ltd., participated in building the hypothesis, performing the experiment and finalizing the manuscript. The authors did not participate in data analysis, interpretation of the data, and the writing of the manuscript.

### Additional information

No additional information is available for this paper.

## Footnotes

1 The “Adonis” function in *R* is used for partitioning distance matrices (i.e., facial shape matrix) among sources of variation and fitting linear models. Statistical significance was tested using a permutation test with pseudo-F ratios.

## References

[1] Bruce V., Young A. (2012). Face Perception.

[2] Calder A., Rhodes G., Johnson M., Haxby J. (2011). Oxford Handbook of Face Perception.

[3] Todorov A., Olivola C.Y., Dotsch R., Mende-Siedlecki P. (2015). Social attributions from faces: determinants, consequences, accuracy, and functional significance. Annu. Rev. Psychol..

[4] Hassin R., Trope Y. (2000). Facing faces: studies on the cognitive aspects of physiognomy. J. Pers. Soc. Psychol..

[5] Todorov A., Porter J.M. (2014). Misleading first impressions: different for different facial images of the same person. Psychol. Sci..

[6] Zebrowitz L.A., Fellous J.-M., Mignault A., Andreoletti C. (2003). Trait impressions as overgeneralized responses to adaptively significant facial qualities: evidence from connectionist modeling. Pers. Soc. Psychol. Rev..

[7] Rhodes G., Simmons L.W., Peters M. (2005). Attractiveness and sexual behavior: does attractiveness enhance mating success?. Evol. Hum. Behav..

[8] Agthe M., Spörrle M., Maner J.K. (2011). Does being attractive always help? Positive and negative effects of attractiveness on social decision making. Pers. Soc. Psychol. Bull..

[9] Little A.C., Burriss R.P., Jones B.C., Roberts S.C. (2007). Facial appearance affects voting decisions. Evol. Hum. Behav..

[10] Rule N., Ambady N. (2010). First impressions of the face: predicting success. Soc. Personal. Psychol. Compass.

[11] Todorov A., Mandisodza A.N., Goren A., Hall C.C. (2005). Inferences of competence from faces predict election outcomes. Science (80-).

[12] Gheorghiu A.I., Callan M.J., Skylark W.J. (2017). Facial appearance affects science communication. Proc. Natl. Acad. Sci. Unit. States Am..

[13] Sutherland C.A.M. (2013). Social inferences from faces: ambient images generate a three-dimensional model. Cognition.

[14] Oosterhof N.N., Todorov A. (2008). The functional basis of face evaluation. Proc. Natl. Acad. Sci. U. S. A..

[15] Todorov A., Dotsch R., Porter J.M., Oosterhof N.N., Falvello V.B. (2013). Validation of data-driven computational models of social perception of faces. Emotion.

[16] Sutherland C.A.M. (2018). Facial first impressions across culture: data-driven modeling of Chinese and British perceivers’ unconstrained facial impressions. Pers. Soc. Psychol. Bull..

[17] Cunningham M.R., Roberts A.R., Barbee A.P., Druen P.B., Wu C.-H. (1995). “Their ideas of beauty are, on the whole, the same as ours”: consistency and variability in the cross-cultural perception of female physical attractiveness. J. Pers. Soc. Psychol..

[18] Jones D., Hill K. (1993). Criteria of facial attractiveness in five populations. Hum. Nat..

[19] Zebrowitz L.A. (2012). First impressions from faces among U.S. and culturally isolated Tsimane’ people in the Bolivian rainforest. J. Cross Cult. Psychol..

[20] Slater A. (1998). Newborn infants prefer attractive faces. Infant Behav. Dev..

[21] Slater A., Quinn P.C., Hayes R., Brown E. (2000). The role of facial orientation in newborn infants’ preference for attractive faces. Dev. Sci..

[22] Cogsdill E.J., Todorov A.T., Spelke E.S., Banaji M.R. (2014). Inferring character from faces: a developmental study. Psychol. Sci..

[23] Wolffhechel K. (2014). Interpretation of appearance: the effect of facial features on first impressions and personality. PloS One.

[24] Walker M., Vetter T. (2009). Portraits made to measure: manipulating social judgments about individuals with a statistical face model. J. Vis..

[25] Perrett D.I. (1998). Effects of sexual dimorphism on facial attractiveness. Nature.

[26] Sell A. (2009). Human adaptations for the visual assessment of strength and fighting ability from the body and face. Proc. Roy. Soc. Lond. B.

[27] Fink B., Neave N., Seydel H. (2007). Male facial appearance signals physical strength to women. Am. J. Hum. Biol..

[28] Sutherland C.A.M. (2015). Personality judgments from everyday images of faces. Front. Psychol..

[29] Bookstein F.L. (1991). Morphometric Tools for Landmark Data: Geometry and Biology.

[30] Abend P., Pflüger L.S., Koppensteiner M., Coquerelle M., Grammer K. (2015). The sound of female shape: a redundant signal of vocal and facial attractiveness. Evol. Hum. Behav..

[31] Boothroyd L.G., Scott I., Gray A.W., Coombes C.I., Pound N. (2013). Male facial masculinity as a cue to health outcomes. Evol. Psychol..

[32] Farrera A., Villanueva M., Quinto-Sánchez M., González-José R. (2015). The relationship between facial shape asymmetry and attractiveness in Mexican students. Am. J. Hum. Biol..

[33] Windhager S., Schaefer K., Fink B. (2011). Geometric morphometrics of male facial shape in relation to physical strength and perceived attractiveness, dominance, and masculinity. Am. J. Hum. Biol..

[34] Kleisner K., Priplatova L., Frost P., Flegr J. (2013). Trustworthy-looking face meets brown eyes. PloS One.

[35] Linke L., Saribay S.A., Kleisner K. (2016). Perceived trustworthiness is associated with position in a corporate hierarchy. Pers. Indiv. Differ..

[36] Třebický V., Havlíček J., Roberts S.C., Little A.C., Kleisner K. (2013). Perceived aggressiveness predicts fighting performance in mixed-martial-arts fighters. Psychol. Sci..

[37] Kleisner K., Chvátalová V., Flegr J. (2014). Perceived intelligence is associated with measured intelligence in men but not women. PloS One.

[38] Komori M., Kawamura S., Ishihara S. (2011). Multiple mechanisms in the perception of face gender: effect of sex-irrelevant features. J. Exp. Psychol. Hum. Percept. Perform..

[39] Mitteroecker P., Windhager S., Müller G.B., Schaefer K. (2015). The morphometrics of “masculinity” in human faces. PloS One.

[40] Lang P.J., Bradley M.M., Cuthbert B.N. (2008). International affective picture system (IAPS): Affective ratings of pictures and instruction manual. Tech. Rep..

[41] Kleisner K., Kočnar T., Rubešová A., Flegr J. (2010). Eye color predicts but does not directly influence perceived dominance in men. Pers. Indiv. Differ..

[42] Rohlf F.J. (2015). The tps series of software. Hystrix.

[43] Windhager S., Bookstein F.L., Millesi E., Wallner B., Schaefer K. (2017). Patterns of correlation of facial shape with physiological measurements are more integrated than patterns of correlation with ratings. Sci. Rep..

[44] Peirce J. (2019). PsychoPy2: experiments in behavior made easy. Behav. Res. Methods.

[45] Bates D., Mächler M., Bolker B., Walker S. (2015). Fitting linear mixed-effects models using lme4. J. Stat. Software.

[46] Ishi H., Gyoba J., Kamachi M., Mukaida S., Akamatsu S. (2004). Analyses of facial attractiveness on feminised and juvenilised faces. Perception.

[47] Said C.P., Sebe N., Todorov A. (2009). Structural resemblance to emotional expressions predicts evaluation of emotionally neutral faces. Emotion.

[48] Zebrowitz L.A. (2017). First impressions from faces. Curr. Dir. Psychol. Sci..

[49] Buss D.M., Schmitt D.P. (1993). Sexual strategies theory: an evolutionary perspective on human mating. Psychol. Rev..

[50] Buss D.M. (1989). Sex differences in human mate preferences: evolutionary hypotheses tested in 37 cultures. Behav. Brain Sci..

[51] Nakamura K., Watanabe K. (2019). Data-driven mathematical model of East-Asian facial attractiveness: the relative contributions of shape and reflectance to attractiveness judgements. R. Soc. Open Sci..

[52] Zebrowitz L.A., Franklin R.G., Hillman S., Boc H. (2013). Older and younger adults’ first impressions from faces: similar in agreement but different in positivity. Psychol. Aging.

[53] Liu S., Fan Y.-Y., Samal A., Guo Z. (2016). Advances in computational facial attractiveness methods. Multimed. Tool. Appl..

[54] Kazemi V., Sullivan J. (2014). One millisecond face alignment with an ensemble of regression trees.

[55] Karras T., Laine S., Aila T. (2019). A style-based generator architecture for generative adversarial networks. Proceedings of the IEEE Conference on Computer Vision and Pattern Recognition.

[56] McCurrie M. (2017). Predicting first iampressions with deep learning. 2017 12th IEEE International Conference on Automatic Face & Gesture Recognition (FG 2017).

[57] Jack R.E., Garrod O.G.B., Yu H., Caldara R., Schyns P.G. (2012). Facial expressions of emotion are not culturally universal. Proc. Natl. Acad. Sci. Unit. States Am..

[58] Leo M. (2018). Computational assessment of facial expression production in ASD children. Sensors (Switzerland).

